# On *Araniella* and *Neoscona* (Araneae, Araneidae) of the Caucasus, Middle East and Central Asia

**DOI:** 10.3897/zookeys.906.47978

**Published:** 2020-01-22

**Authors:** Alireza Zamani, Yuri M. Marusik, Anna Šestáková

**Affiliations:** 1 Zoological Museum, Biodiversity Unit, University of Turku, FI-20014, Finland University of Turku Turku Finland; 2 Institute for Biological Problems of the North RAS, Portovaya Str.18, Magadan, Russia Institute for Biological Problems of the North, Russian Academy of Sciences Magadan Russia; 3 Department of Zoology & Entomology, University of the Free State, Bloemfontein 9300, South Africa University of the Free State Bloemfontein South Africa; 4 The Western Slovakian Museum, Múzejné nám. 3, 918 09 Trnava, Slovakia The Western Slovakian Museum Trnava Slovakia

**Keywords:** Aranei, new species, new combination, new record, new synonymy, orb-web spiders, redescription

## Abstract

New taxonomic data for species belonging to *Araniella* Chamberlin & Ivie, 1942 and *Neoscona* Simon, 1864 occurring in the Caucasus, Middle East and Central Asia are provided. Three species are described as new to science: *A. mithra***sp. nov.** (♂♀, northwestern, central and southwestern Iran), *A. villanii***sp. nov.** (♂♀, southwestern Iran, eastern Kazakhstan and northern India) and *N. isatis***sp. nov.** (♂♀, central Iran). *Neoscona spasskyi* (Brignoli, 1983) **comb. nov., stat. res.** is removed from the synonymy of *N. tedgenica* (Bakhvalov, 1978), redescribed and recorded from Iran and Turkmenistan for the first time. New combinations are established for this species, as well as for *Araniella nigromaculata* (Schenkel, 1963) **comb. nov.** (♀, north-central China) (both ex. *Araneus*). Two new synonymies are proposed: *Araniella tbilisiensis* Mcheidze, 1997 **syn. nov.** is synonymized with *A. opisthographa* (Kulczyński, 1905), and *Neoscona sodom* Levy, 1998 **syn. nov.** is synonymized with *N. theisi* (Walckenaer, 1841); the latter is recorded from Iran, Georgia, and Russia (Northern Caucasus) for the first time.

## Introduction

Araneidae Clerck, 1757 with 3072 valid species (WSC 2019) is the third largest family of spiders. At least in the Palaearctic, it is the best-studied family of spiders due to numerous publications dealing with the survey of regional fauna, or revisions of European and Far East (China, Japan, Korea) species. However, the Central Palaearctic is not well studied in comparison to other parts. Several species described by Bakhvalov (1970, 1974, 1978, 1981) remain known only from the original publications supplied with very schematic figures and brief descriptions. In order to fill this gap, we decided to study all available material from Iran and Central Asian countries and provide step by step reviews of different genera. Among material examined, we recognized two new species of *Araniella* Chamberlin & Ivie, 1942 and one new species of *Neoscona* Simon, 1864. While comparing new species with species occurring in the region, we recognized two new synonyms and two new combinations in both genera. The goals of this paper are to provide illustrated descriptions of new species and redescriptions of poorly known species, along with new combinations, synonymies, and distribution records.

## Materials and methods

Specimens were photographed using an Olympus Camedia E-520 camera attached to an Olympus SZX16 stereomicroscope or to the eye piece of an Olympus BH2 transmission microscope, and a JEOL JSM-5200 scanning electron microscope (SEM) at the Zoological Museum of University of Turku, Finland. Digital images were prepared using CombineZP image stacking software. Illustrations of internal genitalia were made after clearing them in a 10% KOH aqueous solution. Lengths of leg segments were measured on the dorsal side. Measurements are provided for leg I only (IV, if missing) and listed as: total length (femur, patella, tibia, metatarsus, tarsus). All measurements are given in millimeters.

Abbreviations not explained in the text: **ALE** – anterior lateral eye, **AME** – anterior median eye, **PLE** – posterior lateral eye, **PME** – posterior median eye.

Depositories: **MHNG** – Muséum d’histoire naturelle, Genève, Switzerland, **MMUE** – Manchester Museum of the University of Manchester, England, **ZMMU** – Zoological Museum of Moscow University, Moscow, Russia, **ZMUT** – Zoological Museum of University of Turku, Finland, **PPC** – A.V. Ponomarev’s personal collection, Rostov on Don, Russia.

## Taxonomy

### Family Araneidae Clerck, 1757

#### Genus Araniella

Taxon classificationAnimaliaAraneaeAraneidae

Chamberlin & Ivie, 1942

CF47587B-9806-5DFC-B5DF-567A03A08532

##### Type species.

*Epeira displicata* Hentz, 1847 from Alabama, USA.

##### Comments.

Currently, this genus includes 12 species distributed exclusively in the Holarctic (WSC 2019). Only two species, the generotype and *A. proxima* (Kulczyński, 1885), are known in both parts of the realm (Palaearctic and Nearctic); all other species are restricted to the Palaearctic. Although the genus has never been the subject of a global revision, it is well studied, and all species are known by both sexes, with the exception of *A. tbilisiensis* (Mcheidze, 1997). This species was described on the basis of both sexes, but the male palp has never been illustrated.

##### Diagnosis.

The genus well differs from all Holarctic genera of Araneidae by large (as long as embolus and terminal apophysis), claw- or spine-like median apophysis directed mesally (vs. not claw- or spine-like but having at least 2 arms).

#### Araniella mithra sp. nov.

Taxon classificationAnimaliaAraneaeAraneidae

5B77D112-B89C-52E2-8A80-E7DE99BA7122

http://zoobank.org/DC0D034A-4554-4C01-B641-D2C6731DE77F

Figs 1A, C ; 2A, B ; 4A, B ; 6A, B ; 7C ; 8C ; 9C ; 10C ; 18

Araniella proxima : Zamani et al. 2017: 58 (misidentification).

##### Type material.

Iran: ***Holotype*** ♂ and ***paratypes*** 1♂ 2♀ (MHNG), Isfahan Province: Nowgahan, 33°11'N, 50°04'E, 22.06.1974 (A. Senglet); 1♀ (MHNG), Falavarian, 32°34'N, 51°31'E, 14.06.1974 (A. Senglet); 2♂12♀ (MHNG), Chaharmahal & Bakhtiari Province: Dimeh, 32°29'N, 50°16'E, 21.06.1974 (A. Senglet); 1♂ 1♀ (MHNG), West Azarbayjan Province: Maku, 39°08'N, 44°30'E, 23.06.1973 (A. Senglet), 1♂ (MMUE), no label.

**Figure 1.**
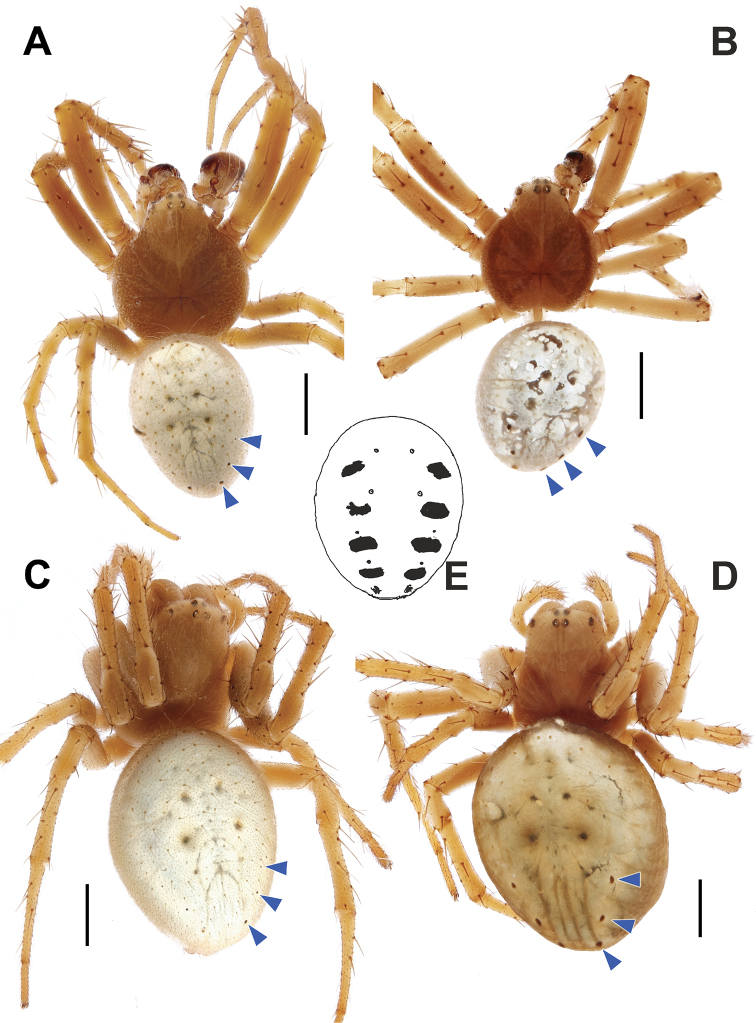
Dorsal habitus of *Araniella mithra* sp. nov. (**A, C**) and *A. villanii* sp. nov. (**B, D**) and abdomen of *A. nigromaculata* (**E**). **A, B** Males **C, D** females. Blue triangles point on black dots on opisthosoma. Scale bars: 1 mm.

**Figure 2.**
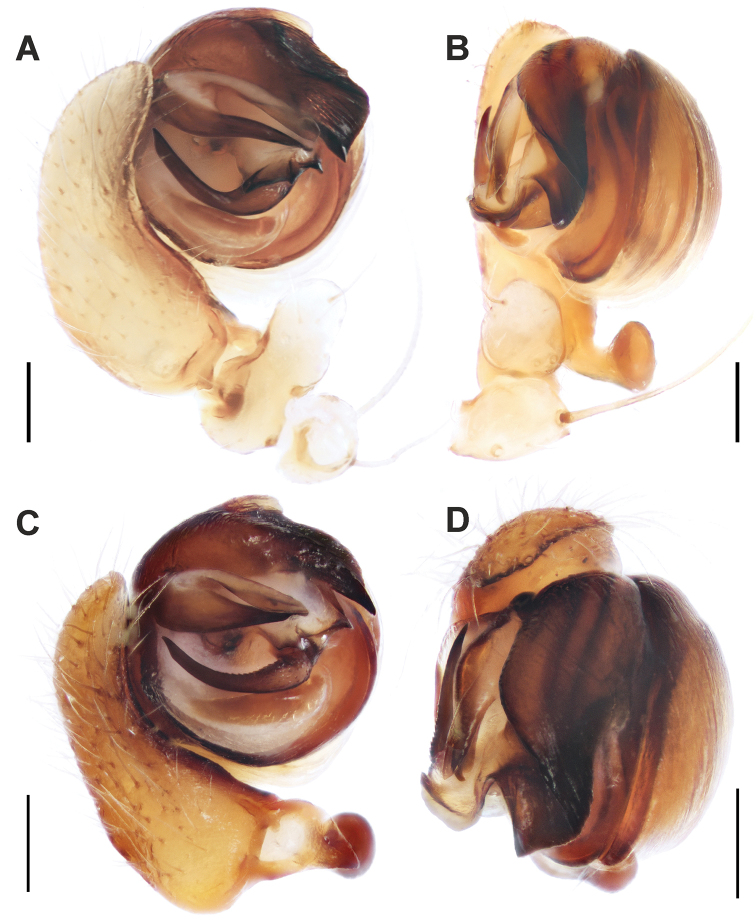
Male palps of *Araniella mithra* sp. nov. (**A, B**) and *A. opisthographa* (**C, D**). **A, C** Retrolateral **B, D** ventral. Scale bars: 0.2 mm.

##### Comparative material.

*Araniella opisthographa* (Kulczyński, 1905). Finland: 1♂ (ZMUT): Åland Islands: Lemland, Rörstorp, 27.06.1971 (P. Lehtinen); Iran: 1♂1♀1sub♂ (ZMMU): Mazandaran Province: Barseh Vil., 36°37'N, 50°41'E, 10.06.2000 (Y.M. Marusik). Turkey: 1♂5♀1sub♂ (ZMMU): Kastamonu Province: Azdavay Dist., 41°41'N, 33°25'E, 975 m, 30.05.2009 (Y.M. Marusik).

##### Etymology.

The specific epithet is a noun in apposition, and refers to Mithra, the god of light in ancient Indo-Iranian mythology.

##### Diagnosis.

Male palp and epigyne resemble those of *A. opisthographa*, but the two species can be differentiated by the following characters: 1) the embolus is slimmer in *A. mithra* sp. nov., vs. triangular-shaped and with a wider base in *A. opisthographa*; 2) the terminal apophysis in *A. mithra* sp. nov. is almost as wide over its entire length, vs. wider near the peak in *A. opisthographa*; 3) the conductor in *A. mithra* sp. nov. has three distinct spikes, vs. one spike and one more rounded process in *A. opisthographa*; 4) the tegulum in *A. mithra* sp. nov. is higher with a short pointed tip, vs. the slender tegulum with a longer tip in *A. opisthographa*; 5) male carapace unicolor in *A. mithra* sp. nov., vs. presence of broad dark marginal bands in *A. opisthographa*; 6) epigyne with slightly longer scape, and the sclerotized bulges are rounded around the base of scape in *A. mithra* sp. nov., vs. more incised triangular bulges in *A. opisthographa*.

##### Description

(colors and pattern seem faded). **Male** (holotype). Habitus as in Fig. 1A. Total length 5.04. Carapace 2.36 long, 2.19 wide in pars thoracica, 0.91 in pars cephalica. Eye sizes and interdistances: AME: 0.09, ALE: 0.09, PME: 0.11, PLE: 0.12, AME–AME: 0.13, PME–PME: 0.12. Carapace, sternum, labium, chelicerae, and maxillae reddish brown, lighter ventrally and in pars cephalica, without any patterns. Legs the same color as the carapace. Abdomen pale (stored in alcohol, most probably green in live specimens) dorsally, dark gray ventrally, with three pairs of black lateral spots on dorsum posteriorly. Spinnerets light brown, apical segment lighter. Leg I measurements: 7.46 (2.21, 0.93, 1.75, 1.75, 0.82).

Palp as in Figs 2A, B; 4A, B; 6A, B. Tegulum with low round ridge and terminally with short pointed tip; terminal apophysis with blunt end and almost equally wide along its length; embolus pointed, sickle-shaped bent; median apophysis sickle-shaped bent upwards, covered by small denticles (less visible via stereomicroscope), with pointed tip ended near base of embolus; conductor with three distinct spikes.

**Female.** Habitus as in Fig. 1C. Total length 5.65. Carapace 2.40 long, 1.87 wide in pars thoracica, 1.19 in pars cephalica. Eye sizes and interdistances: AME: 0.11, ALE: 0.12, PME: 0.12, PLE: 0.09, AME–AME: 0.14, PME–PME: 0.11. Coloration as in male, slightly lighter. Leg I measurements: 7.30 (2.08, 1.02, 1.60, 1.75, 0.85).

Epigyne as in Figs 7C, 8C, 9C, 10C. Scape longer than wide, slightly wider at its base, reaching distinctly beyond epigyne. Copulatory ducts visible through epigynal cuticle. Receptacles oval, entrance ducts touching each other. Median plate (posterior view), between lateral sclerotized copulatory bulges, round and widest in its center.

##### Phenology.

Adult males and females were collected in mid and late June.

##### Distribution.

Known only from the type localities in northwestern, central and southwestern Iran. It is possible that some of the previous Iranian records of *A. opisthographa* refer to this species.

#### Araniella villanii sp. nov.

Taxon classificationAnimaliaAraneaeAraneidae

72BF7FAB-8383-5E51-AB69-EBA32E35590E

http://zoobank.org/067356F5-0F8F-4F5D-A3D9-604EE3AEDC12

Figs 1B, D ; 3A, B ; 4C, D ; 5A, B ; 7A ; 8A ; 9A ; 10A ; 18

##### Type material.

Iran: ***Holotype*** ♂ and ***paratypes*** 1♀ (MHNG), Chaharmahal & Bakhtiari Province: Kuhrang, 32°28'N, 50°08'E, 19.06.1974 (A. Senglet). Kazakhstan: 2♂ 4♀ (ZMMU), East Kazakhstan Region: Urzhar Distr., Tarbagatai Mt. Range, 5 km NE of Alekseevka, Urzharka river canyon, left bank, 47°17'N, 81°37'E, 1050–1200 m, 23.06.2001 (A.V. Gromov); 3♂ 4♀ (ZMMU), Urzhar Distr., 7–8 km NE of Karatuma [=Kirovka], Tarbagatai Mt. Range, Sholakterek river canyon, left bank, 47°10'N, 82°06'E, 1200–1250 m, 23.06.2001 (A.V. Gromov); 1♂ 2♀ (ZMMU), Urzhar Distr., ca. 4 km NE of Kyzylbulak [=Petrovskoye], Kyzylbulak river canyon, left bank, 47°03'N, 82°18'E, 1100–1150 m, 21.06.2001 (A.V. Gromov). India: 6♂ 2♀ (MMUE), Himachal Pradesh State: Tandi Vill., 5 km S of Keylong, 2700 m, 11.06.1999 (Y.M. Marusik); 1♂ 1♀ (MMUE), Jahalman Vill., 32°38'N, 76°51'E, 3000–3100 m, 13.06.1999 (Y.M. Marusik).

**Figure 3.**
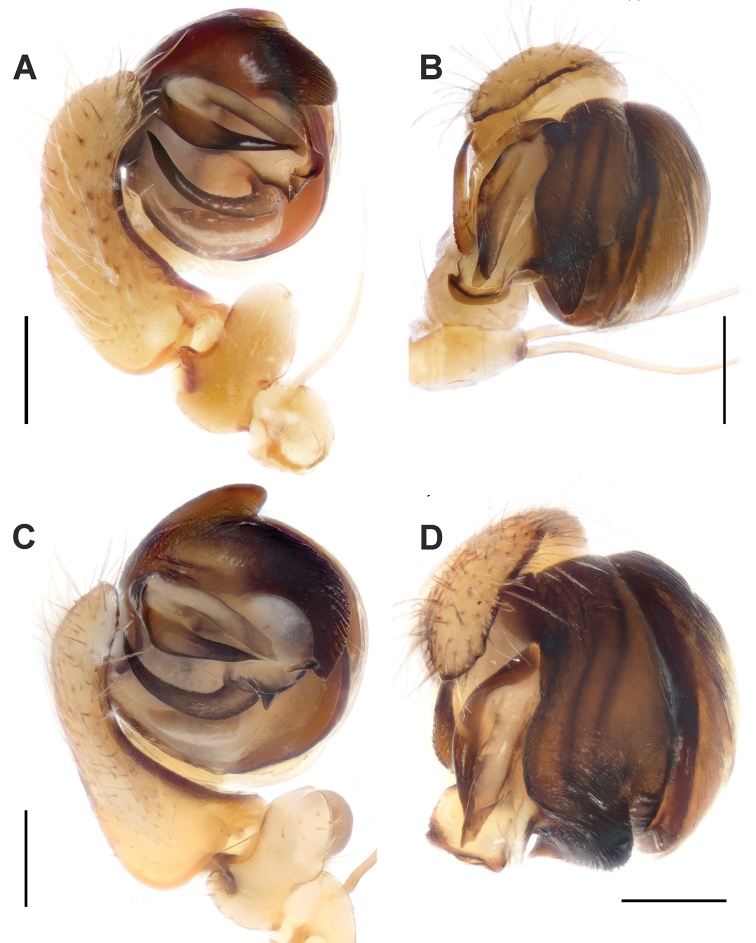
Male palps of *Araniella villanii* sp. nov. (**A, B**) and *A. proxima* (**C, D**). **A, C** Retrolateral **B, D** ventral. Scale bars: 0.2 mm.

**Figure 4.**
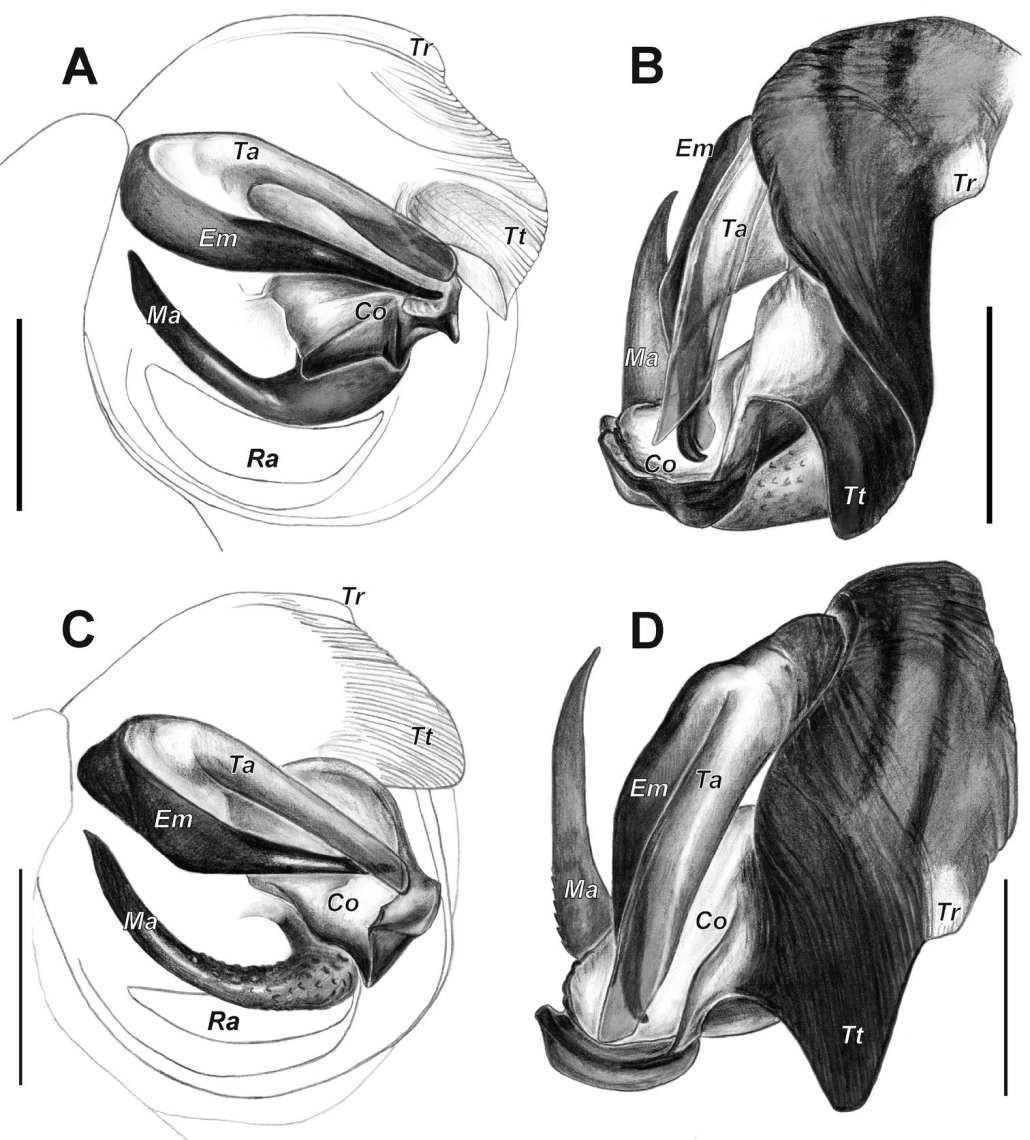
Male palps of *Araniella mithra* sp. nov. (**A, B**) and *A. villanii* sp. nov. (**C, D**). **A, C** Retrolateral **B, D** ventral. Abbreviations: *Co* conductor, *Em* embolus, *Ma* median apophysis, *Ra* radix, *Ta* terminal apophysis, *Tr* tegular ridge, *Tt* tip of tegulum. Scale bars: 0.2 mm.

**Figure 5.**
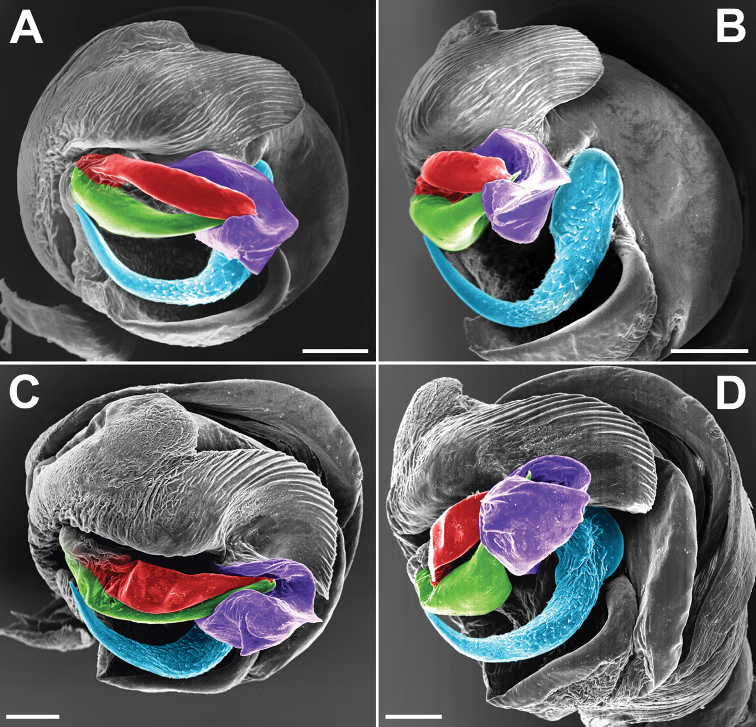
SEM graphs of the bulbs of *Araniella villanii* sp. nov. (**A, B**) and *A. proxima* (**C, D**). **A, C** Retrolateral **B, D** ventro-retrolateral. Blue – median apophysis, green – embolus, red – terminal apophysis, violet – conductor. Scale bars: 0.1 mm.

##### Comparative material.

*Araniella proxima* (Kulczyński, 1885). Russia: 1♂ 1♀ (ZMMU): SE Tuva, Tere-Khol Lake, Sharlaa Stand and vicinity, 50°01'N, 95°03'E, 1050 m, 6–14.07.1996 (Y.M. Marusik).

##### Etymology.

This species is named after French mathematician Cédric Villani (born 5.10.1973), winner of the Fields Medal in 2010 and the former director of Sorbonne University’s Henri Poincaré Institute, for his “mysterious love” for spiders.

##### Diagnosis.

Male palp and epigyne resemble those of *A. proxima* and *A. opisthographa*. Both species, compared to *A. villanii* sp. nov., have similar shape of embolus, and terminal apophysis is identical to that of *A. proxima* and conductor is identical to that of *A. opisthographa*. However, the new species can be diagnosed by the following characteristics: 1) the tegulum in *A. villanii* sp. nov. is markedly shorter, higher, protruding and rounded, vs. more compact non-protruding tegulum with distinctly higher ridge in *A. proxima*, and slender with pointed tip in *A. opisthographa*; 2) the terminal apophysis in *A. villanii* sp. nov. is almost as wide throughout its length, vs. wider at the tip in *A. opisthographa*; 3) the conductor in *A. villanii* sp. nov. has one spike and one more rounded process connected to each other, vs. two independent spikes in *A. proxima*; 4) the median apophysis in *A. villanii* sp. nov. is longer in comparison to both mentioned species; 5) epigyne of *A. villanii* sp. nov. has a distinctly broader scape, vs. slender in *A. proxima* and *A. opisthographa*; 6) the median plate is narrower and more rectangular in the new species, vs. wider and rounded plate in *A. opisthographa* and triangular plate in *A. proxima*; 7) receptacles and entrance ducts in *A. villanii* sp. nov. do not touch each other, but in *A. opisthographa* both structures touch each other, and in *A. proxima* only receptacles touch each other.

##### Description

(colors and pattern seem faded). **Male** (holotype). Habitus as in Fig. 1B. Total length 4.37. Carapace 1.91 long, 1.69 wide in pars thoracica, 0.76 in pars cephalica. Eye sizes and interdistances: AME: 0.08, ALE: 0.07, PME: 0.09, PLE: 0.09, AME–AME: 0.12, PME–PME: 0.11. Carapace, sternum, labium, chelicerae, and maxillae reddish brown, lighter ventrally, carapace with two broad dark marginal bands. Legs lighter in color than the carapace, distally with dark broad annulations. Abdomen pale (stored in alcohol, most probably green in live specimens) dorsally, dark gray ventrally, posterodorsally with three pairs of black lateral spots. Spinnerets light brown, apical segment lighter. Leg I measurements: 6.43 (1.97, 0.82, 1.50, 1.46, 0.68).

Palp as in Figs 3A, B; 4C, D; 5A, B. Tegulum terminally blunt with round ridge; terminal apophysis with blunt end and almost equally wide along its length; embolus triangular-shaped, with wider base; median apophysis sickle-shaped bent upwards with pointed tip ending near base of embolus and covered by many small denticles; conductor with one distinct spike and one more rounded process.

**Female.** Habitus as in Fig. 1D. Total length 6.00. Carapace 2.58 long, 2.15 wide in pars thoracica, 1.29 in pars cephalica. Eye sizes and interdistances: AME: 0.09, ALE: 0.08, PME: 0.10, PLE: 0.09, AME–AME: 0.14, PME–PME: 0.12. Coloration as in male. Leg I measurements: 6.78 (1.93, 0.98, 1.49, 1.51, 0.87).

Epigyne as in Figs 7A, 8A, 9A, 10A. Scape wider in the middle, extending beyond epigynal plate. Copulatory ducts not clearly visible through epigyne cuticle. Oval receptacles are about half their diameter apart; entrance ducts a similar distance apart. Median plate (posterior view), between lateral sclerotized copulatory bulges, slender, slightly wider in the middle.

##### Phenology.

All adult specimens were collected in mid and late June.

##### Distribution.

Known only from the type localities in southwestern Iran, eastern Kazakhstan and northern India. Potentially widely distributed in the Middle East and Central Asia.

#### Araniella opisthographa

Taxon classificationAnimaliaAraneaeAraneidae

(Kulczyński, 1905)

327F5EAD-E551-575C-A9D0-C2BACC9E9A32

**Figure 6.**
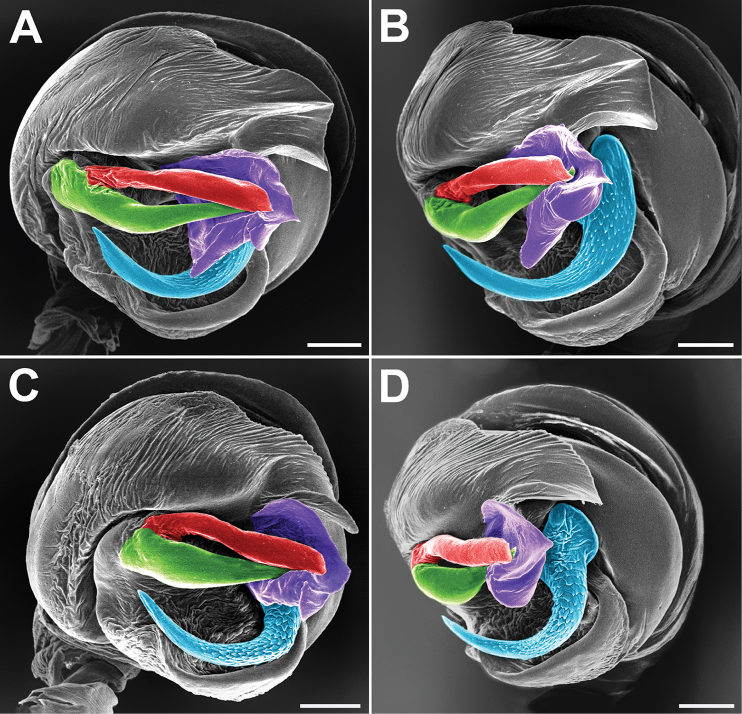
SEM graphs of the bulbs of *Araniella mithra* sp. nov. (**A, B**) and *A. opisthographa* (**C, D**). **A, C** Retrolateral **B, D** ventro-retrolateral. Blue – median apophysis, green – embolus, red – terminal apophysis, violet – conductor. Scale bars: 0.1 mm.

**Figure 7.**
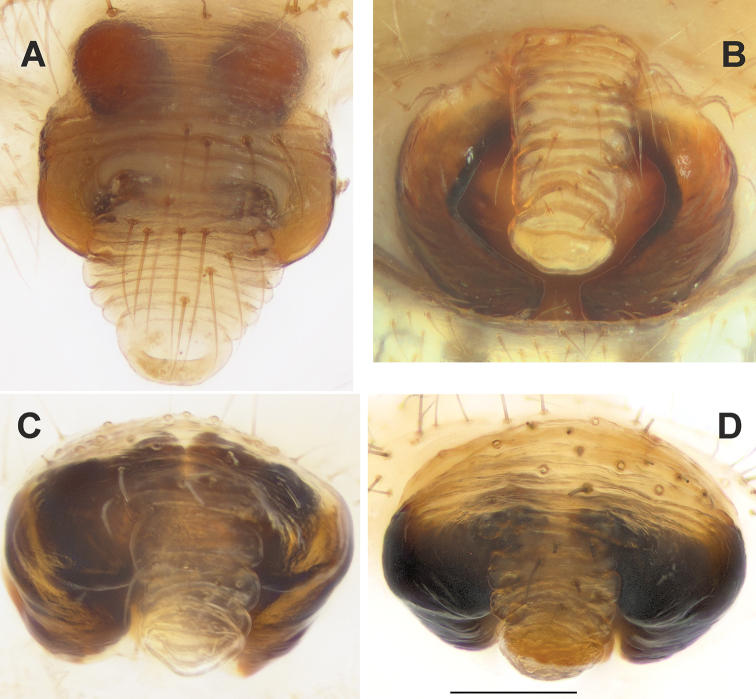
Ventral view of epigynes of *Araniella villanii* sp. nov. (**A**), *A. proxima* (**B**), *A. mithra* sp. nov. (**C**) and *A. opisthographa* (**D**). Scale bar: 0.2 mm.

**Figure 8.**
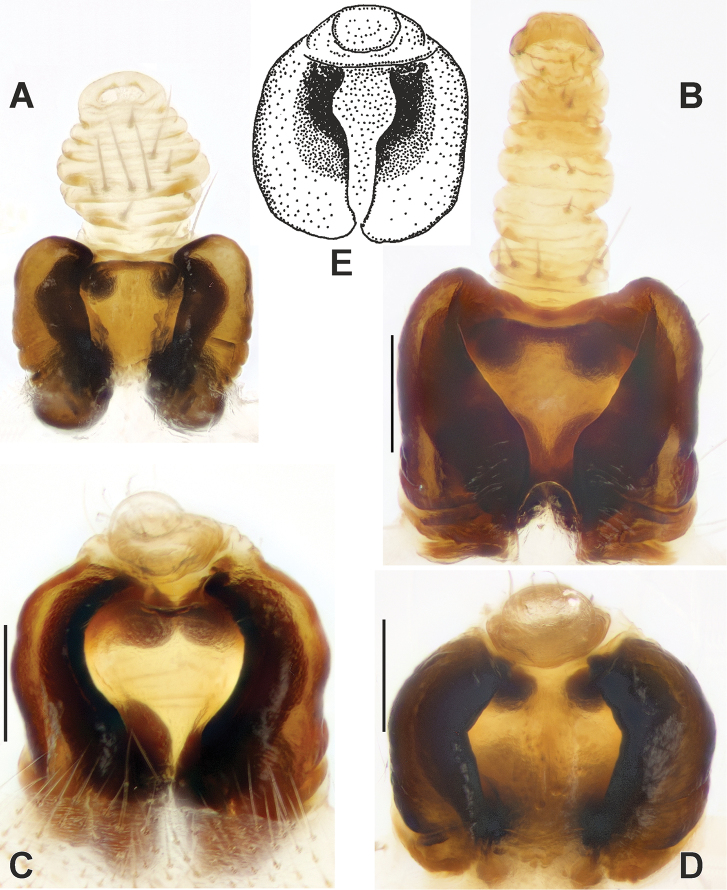
Posterior view of epigynes of *Araniella villanii* sp. nov. (**A**), *A. proxima* (**B**), *A. mithra* sp. nov. (**C**), *A. opisthographa* (**D**) and *A. nigromaculata* (**E**). Scale bars: 0.2 mm.

**Figure 9.**
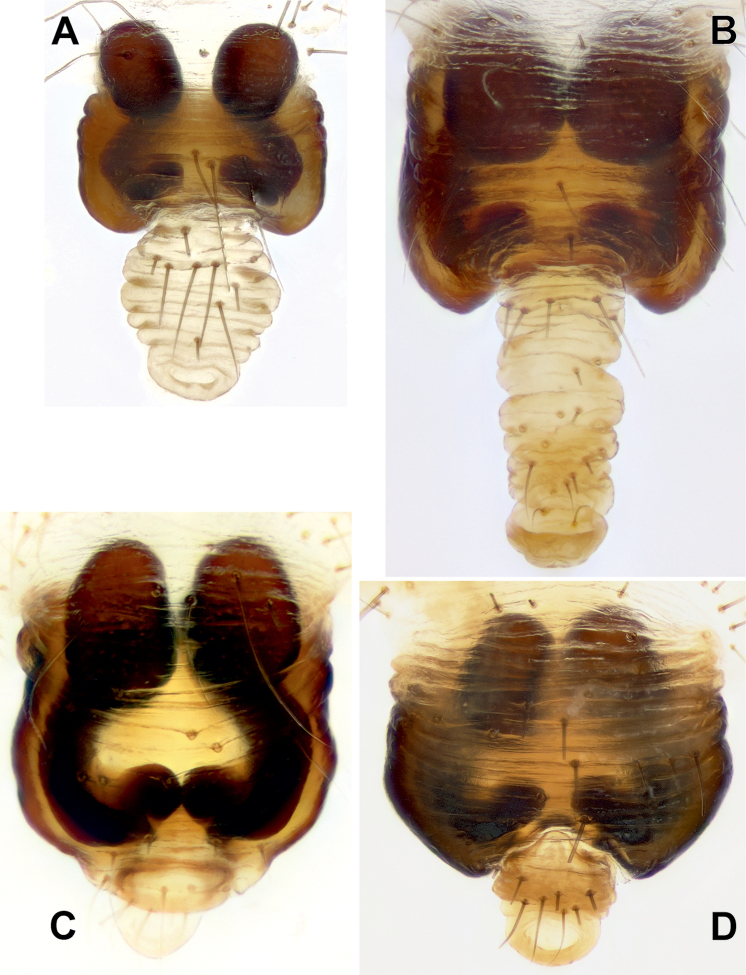
Anterior view of epigynes of *Araniella villanii* sp. nov. (**A**), *A. proxima* (**B**), *A. mithra* sp. nov. (**C**) and *A. opisthographa* (**D**).

**Figure 10.**
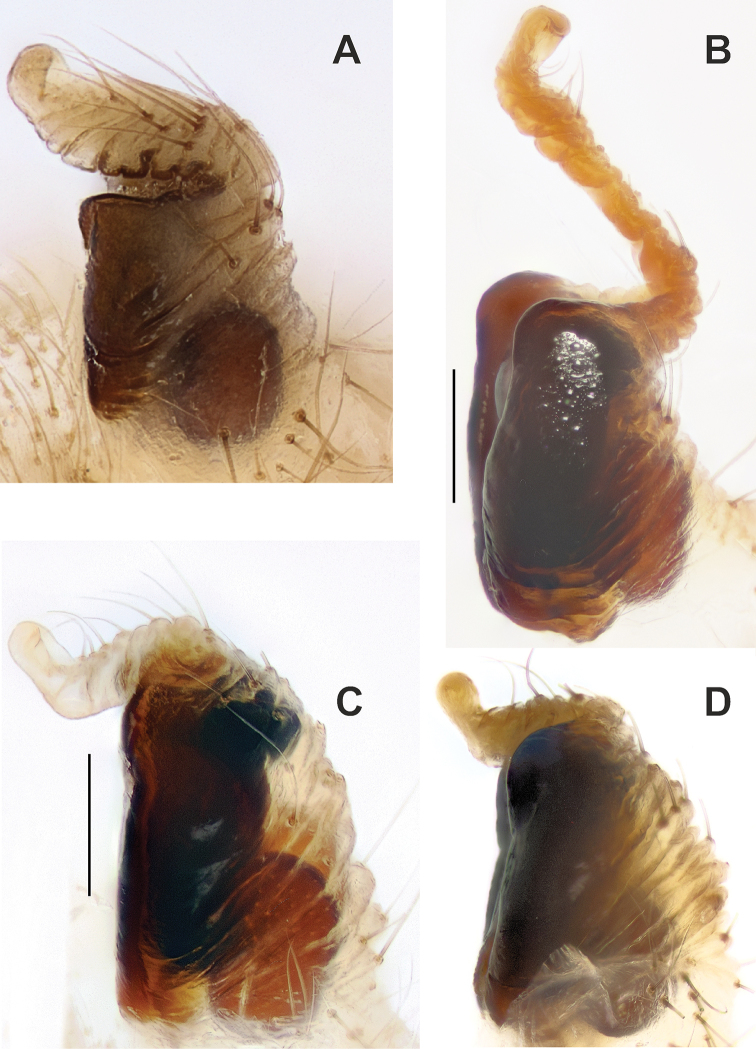
Lateral view of epigynes of *Araniella villanii* sp. nov. (**A**), *A. proxima* (**B**), *A. mithra* sp. nov. (**C**) and *A. opisthographa* (**D**). Scale bars: 0.2 mm.

Figs 2C, D ; 6C, D ; 7D ; 8D ; 9D ; 10D

Araniella opisthographa : Blanke 1982: 289, fig. 3c–d, 5c–d, 6c–d, 8b (♂♀); Roberts 1995: 328, fig. (♂♀); Almquist 2005: 154, fig. 162a–g (♂♀).
Araneus tbilisiensis Mcheidze, 1997: 280, fig. 642–644 (♂♀). **syn. nov.**

##### Comments.

*Araneus tbilisiensis* was described based on one male and four females from the environs of Tbilisi, Georgia. There is no indication which specimen/sex was selected as the holotype. Mcheidze (1997) provided figures of male and female habitus, as well as epigyne, but the male palp was not illustrated. Judging from the figure of epigyne and distribution, it is most likely a junior synonym of *A. opisthographa*, which is already known from the surroundings of Tbilisi (Otto 2019). We tried to obtain the type material for this study, but we have been informed that the single male specimen is most probably lost (V. Pkhakadze, pers. comm.).^*^

#### Araniella nigromaculata

Taxon classificationAnimaliaAraneaeAraneidae

(Schenkel, 1963) comb. nov.

D87105AC-CC38-5E32-A1B2-54F78D79F2EC

Figs 1E , 8E

Araneus nigromaculatus Schenkel, 1963: 154, fig. 91a–c (♀).
Araneus nigromaculatus : Yin et al. 1997: 204, fig. 122a–c (♀); Song et al. 1999: 240, fig. 139e, f, 148l (♀).

##### Comments.

The female holotype was collected in southern Gansu (ca. 33°40'N, 104°20'E), north-central China. Figures of Yin et al. (1997) and Song et al. (1999) are reproduced after Schenkel (1963). The holotype (in Muséum National d’Histoire Naturelle, Paris) was examined in 1980 by Yuri Marusik and illustrated, but no data have been copied from the label. Abdominal pattern and shape of epigyne indicates its belonging to *Araniella* and therefore we provide a new combination.

#### Genus Neoscona

Taxon classificationAnimaliaAraneaeAraneidae

Simon, 1864

CEBF95FB-EE61-51E4-91BA-B671C2CBA151

Neoscona Simon, 1864: 261.
Neoscona : Berman and Levi 1971: 469; Grasshoff 1986: 4; Tanikawa 1998: 134.

##### Type species.

*Epeira arabesca* Walckenaer, 1841, fixed by F. O. Pickard-Cambridge 1904: 466.

##### Note.

Simon (1864) proposed this genus for nine species currently considered in *Larinioides* Caporiacco, 1934, *Araneus* Clerck, 1757 and *Neoscona*. Although type species were not fixed for any genera described in Simon’s book [there were no rules for type fixation at that time], the author used the term ‘espèces principales’ (=main species). Simon (1864) considered “L’*épéire scalaire* (*neoscona*)” (=*A. marmoreus* Clerck, 1757) as the “main species”.

##### Comments.

With 124 valid species (WSC 2019), *Neoscona* is the third largest genus in Araneidae. Only *Araneus* Clerck, 1757 (595 spp.) and *Cyclosa* Menge, 1866 (180 spp.) are more speciose. At the same time, it has the highest number of synonyms (114) and *nomina dubia* (10) (WSC 2019) in comparison to the valid names. The genus has an almost global distribution, unknown only in South America. It is relatively well studied in North America, Africa, China, and Japan due to the revisions by Berman and Levi (1971), Grasshoff (1986), Yin et al. (1997) and Tanikawa (1998), respectively, but remains poorly known in the Central Asia, India, South East Asia and Australia. Although the male palp is rather uniform in shape across the genus, epigynes can be split into two morphotypes, with inflexible scape (*Neoscona* s. str.) and with flexible scape (*Afraranea* Archer, 1951, a genus currently considered as a synonym of *Neoscona* in WSC (2019) with reference to Grasshoff (1986), although the latter author considered *Afraranea* as a subgenus of *Neoscona*).

Currently, six species of *Neoscona* are known in the region: *N. adianta* (Walckenaer, 1802), *N. subfusca* (C.L. Koch, 1837), *N. theisi* (Walckenaer, 1841) (all throughout the region), *N. spasskyi* (Brignoli, 1983) (Tajikistan, Kyrgyzstan, Turkmenistan, Iran), *N. tedgenica* (Bakhvalov, 1978) (Turkmenistan) and *N. isatis* sp. nov. (Iran).

#### Neoscona adianta

Taxon classificationAnimaliaAraneaeAraneidae

(Walckenaer, 1802)

9CA08845-DA1B-5996-8BBF-D0075DAA054A

Neoscona adiantum : Grasshoff 1986: 66, fig. 85–89 (♂♀).
Neoscona adianta : Levy 1998: 339, fig. 108–116 (♂♀); Tanikawa 1998: 140, fig. 9, 18–24 (♂♀); Tanikawa 2007: 68, fig. 160–161, 575–577 (♂♀).

##### Diagnosis.

Both sexes of this species well differ from other congeners occurring in Central Asia, Iran and Caucasus by the absence of a white median band on the sternum.

##### Description.

See above-cited literature.

##### Distribution.

Transpalaearctic, known throughout the region: Armenia, Azerbaijan, Georgia, Iran, Turkmenistan, Uzbekistan, Kazakhstan, Kyrgyzstan, Tajikistan, and Altai in South Siberia (Mikhailov 2013, Zamani et al. 2019).^*^

#### Neoscona isatis sp. nov.

Taxon classificationAnimaliaAraneaeAraneidae

24DF877C-81AF-55D7-9A8E-691650A0D0E5

http://zoobank.org/06094E25-00A6-473B-9AC7-4319CD43F833

**Figure 11.**
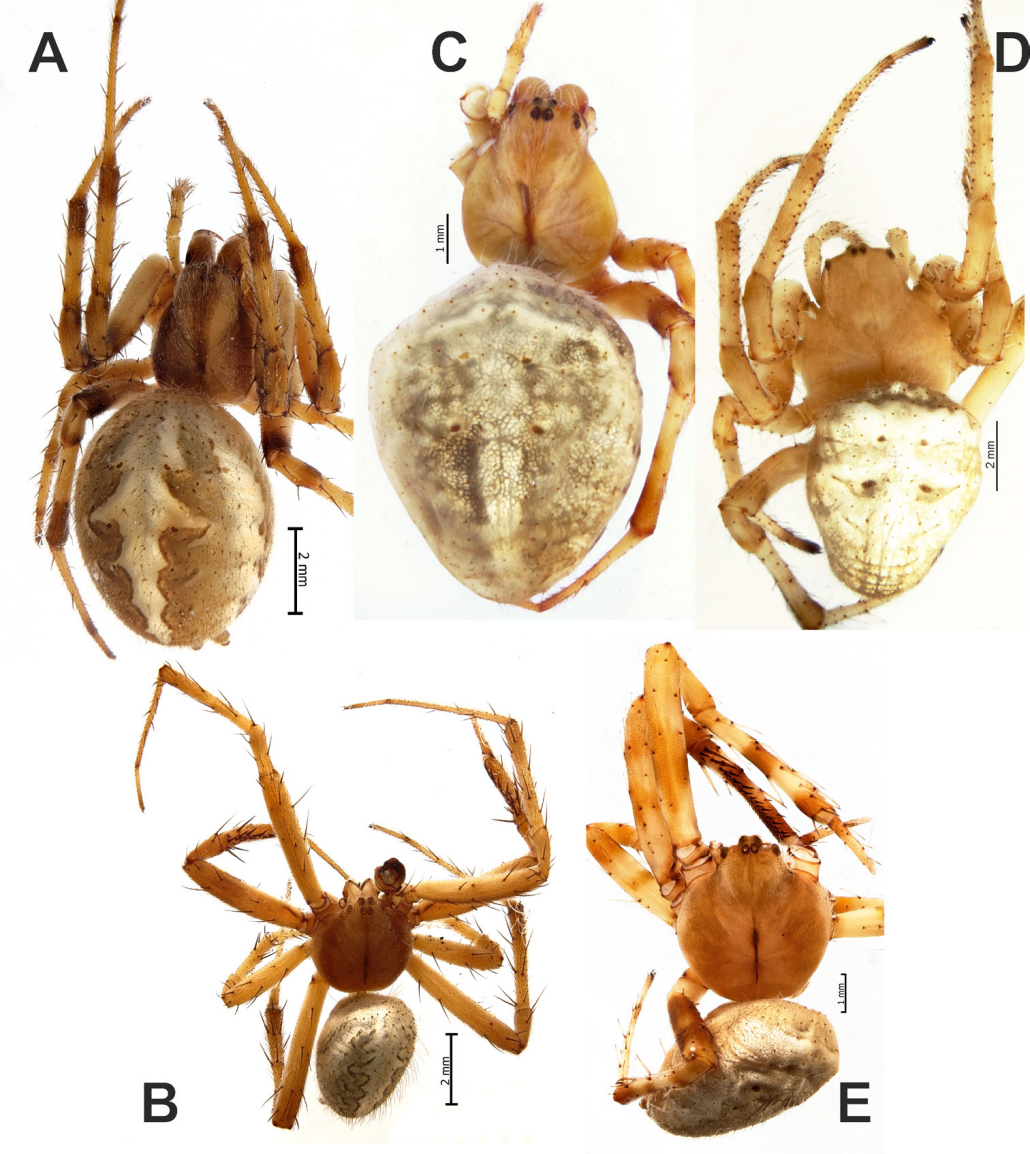
Dorsal habitus of *Neoscona theisi* (**A, B**), *N. spasskyi* (**C**) and *N. isatis* sp. nov. (**D, E**). **A, C, D** Females **B, E** males.

Figs 11D, E ; 13E, F ; 14B, F ; 15E–G ; 16D–F ; 17G–I ; 18

##### Type material.

Iran: ***Holotype*** ♂ and ***paratype*** 1♀ (MHNG), Yazd Province: Ahmadabad, 32°20'N, 53°59'E, 15.08.2018 (A. Zamani).

##### Etymology.

The specific epithet is a noun in apposition, and refers to the historic name of Yazd, the type locality of the species.

##### Diagnosis.

The new species is similar to *N. theisi* and *N. spasskyi* in having a white median band on sternum (Fig. 13A, C, E), but well differs by having a broad white median band on the venter of abdomen (vs. venter with lateral white band, and dark median band). Males of *N. isatis* sp. nov. can be easily distinguished from the species occurring in the region by numerous small spines on tibia II (Fig. 14F) lacking in other species (Fig. 14D–E) and median apophysis lacking prolateral extension (*Me*) (vs. present). Epigyne of this species well differs from other species occurring in Central Asia by having prominent lateral extensions (*Le*) as long as wide and long scape (*Sc*) almost 2 times longer than wide (vs. lateral extensions absent or poorly developed and scape almost as wide as long, cf. Fig. 17A, D, G).

##### Description.

**Male.** Habitus as in Fig. 11E. Total length 9.62. Carapace 4.14 long, 3.90 wide in pars thoracica, 1.28 in pars cephalica. Eye sizes and interdistances: AME: 0.20, ALE: 0.19, PME: 0.14, PLE: 0.14, AME–AME: 0.25, PME–PME: 0.10. Carapace, labium, chelicerae, and maxillae light brown, carapace with distinct and relatively long foveal mark, slightly darker in submarginal and without any patterns. Sternum with light median band. Legs the same color as the carapace, with annulations and numerous spines. Tibia II ventrally with about 90 spines of three types, fine – over 50, medium-sized – over 30, and few macrospines. Abdomen light yellowish, with scattered long white setae, dorsally with a horizontal gray line anteriorly, and a gray longitudinal branched pattern medially, with a brown dot on each side; ventrally with a white patch between epigastric furrow and spinnerets area. Spinnerets light brown, apical segment lighter. Leg IV (leg I incomplete) measurements: 14.39 (4.84, 1.91, 3.07, 3.47, 1.10).

Palp as in Figs 14B, 15E–G, 16D–F. Tegulum without distinct ventral extension; median apophysis (*Ma*) without prolateral extension, stipes of median apophysis (*Sm*) as long as apophysis; lamella (*La*) weakly sclerotized; conductor club-like.

**Female.** Habitus as in Fig. 11D. Total length 11.56. Carapace 5.02 long, 3.49 wide in pars thoracica, 1.74 in pars cephalica. Eye sizes and interdistances: AME: 0.17, ALE: 0.17, PME: 0.18, PLE: 0.19, AME–AME: 0.31, PME–PME: 0.12. Coloration generally as in male, slightly lighter and more uniform, with less distinct patterns and markings, and abdomen with an additional two brown dots on dorsum, without any distinct patterns. Leg I measurements: 18.11 (5.01, 2.69, 4.27, 4.53, 1.61).

Epigyne as in Figs 13F, 17G–I. Long, with scape (*Sc*) as long as base; lateral extensions (*Le*) prominent, as long as wide, originates dorsally; scape almost twice longer than wide.

##### Distribution.

Known only from the type locality in Yazd Province, central Iran.

#### Neoscona spasskyi

Taxon classificationAnimaliaAraneaeAraneidae

(Brignoli, 1983), comb. nov., stat. res.

F8986B96-73BA-50CD-8B91-AA463CFB76FA

Figs 11C ; 12A, B ; 13C–D ; 14C, E ; 15A–C ; 16A–C ; 17D–F ; 18

Araneus cruciferoides Spassky, 1952: 203, fig. 6, 10 (♂♀).
Araneus spasskyi : Brignoli 1983: 258 (replacement name for A. cruciferoides).
Neoscona tedgenica : Marusik et al. 1991: 20 (misidentified).

##### Material examined.

Iran: 1♂ 3♀ (ZMMU): Golestan Province: Ramiyan, 36°59'N, 55°07'E, 29.07.74 (A. Senglet); 1♀ (MHNG): Razavi Khorasan Province: route to Amirabad, 36°47'N, 59°54'E, 1100 m, 23.07.74 (A. Senglet); 3♂ 9♀ (MHNG): North Khorasan Province: Bojnurd, 37°29'N, 57°26'E, 26.07.74 (A. Senglet); Turkmenistan: 1♂ 2♀ (ZMMU): Balkan Province: Magtymguly (formerly Garrygala, Kara-Kala), in house, 02.08.79 (V. Fet).

**Figure 12.**
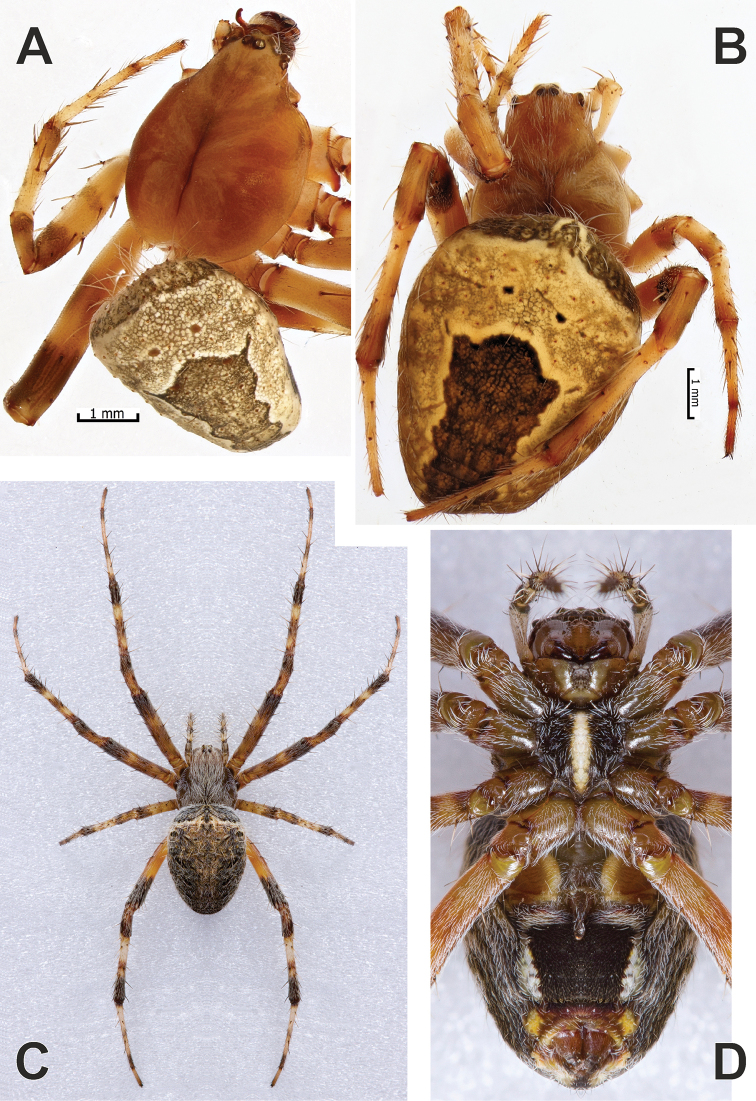
Habitus of *Neoscona spasskyi* (**A, B**) and *N. theisi* (**C, D**). **A–C** Dorsal **D** ventral **C, D** showing variations in comparison to specimens depicted in Figure 11. Photos **C, D** courtesy of A. Seropian.

**Figure 13.**
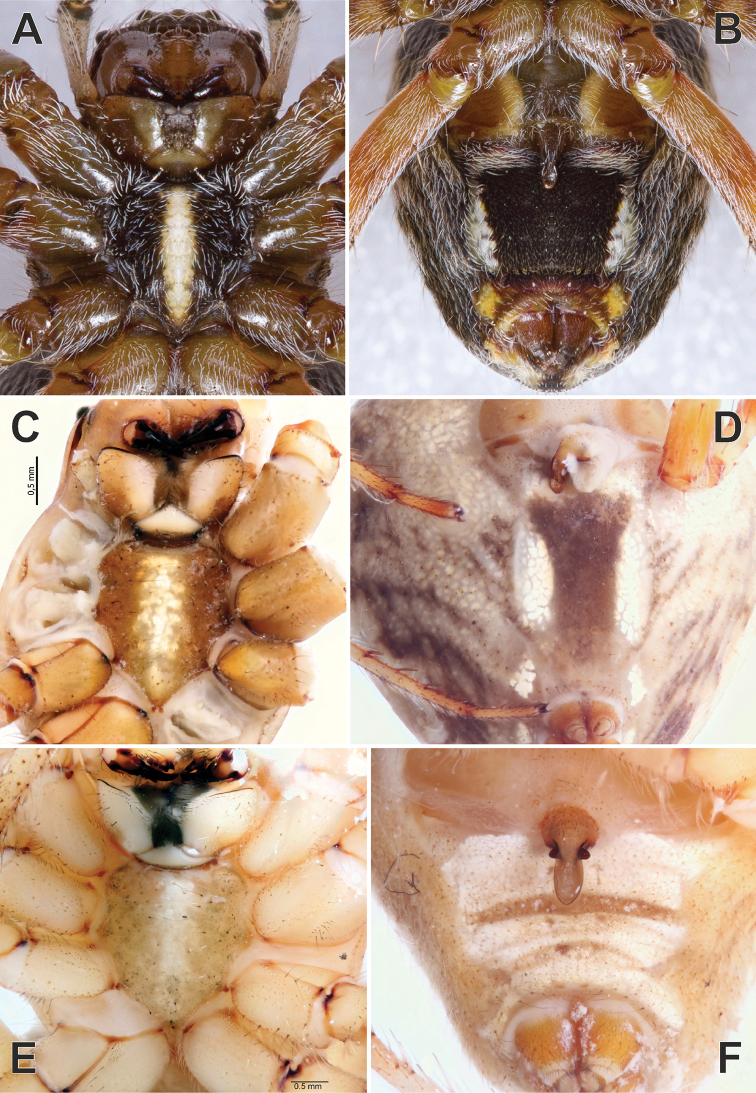
Females of *Neoscona theisi* (**A, B**), *N. spasskyi* (**C, D**) and *N. isatis* sp. nov. (**E, F**). **A, C, E** Prosoma, ventral **B, D, F** abdomen, ventral. Photos **A, B** courtesy of A. Seropian.

**Figure 14.**
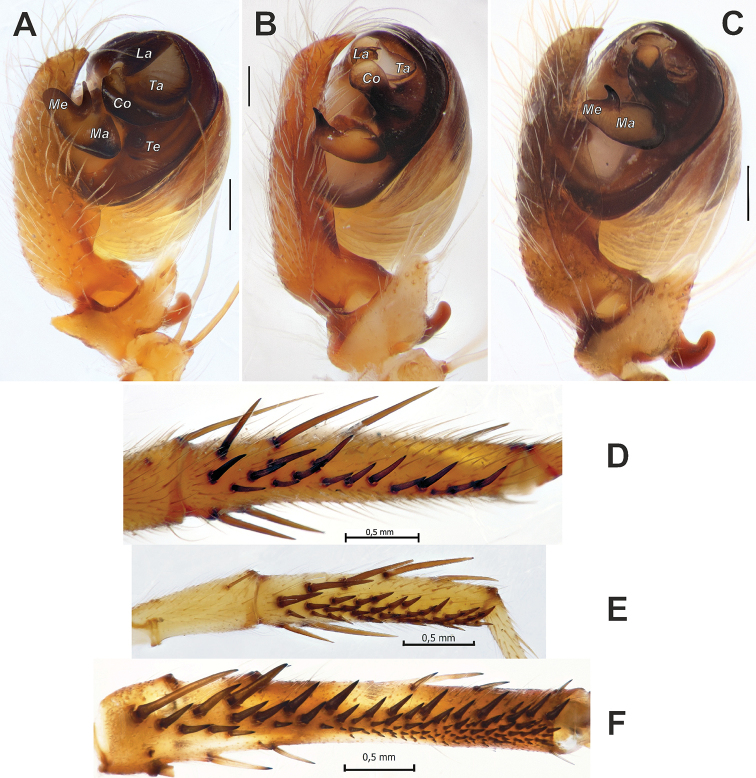
Male palps and tibiae II of *Neoscona theisi* (**A, D**), *N. isatis* sp. nov. (**B, F**) and *N. spasskyi* (**C, E**). **A–C** Male palp, prolateral **D–F** male tibia II, ventral. Abbreviations: *Co* conductor, *La* lamella, *Ma* median apophysis, *Me* extension of median apophysis, *Ta* terminal apophysis, *Te* tegulum. Scale bars: 0.2 mm, unless stated otherwise.

**Figure 15.**
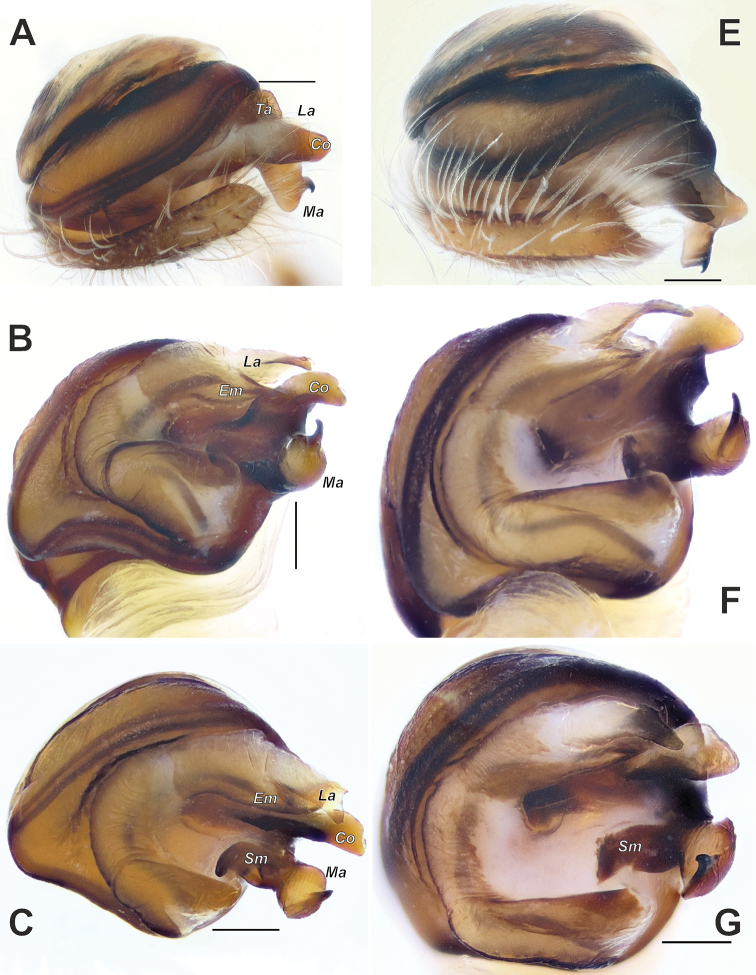
Male palps of *Neoscona spasskyi* (**A–C**) and *N. isatis* sp. nov. (**E–G**). **A, C, E, G** Anterior **B, F** ventral. Abbreviations: *Co* conductor, *Em* embolus, *La* lamella, *Ma* median apophysis, *Sm* stipes of median apophysis, *Ta* terminal apophysis. Scale bars: 0.2 mm.

**Figure 16.**
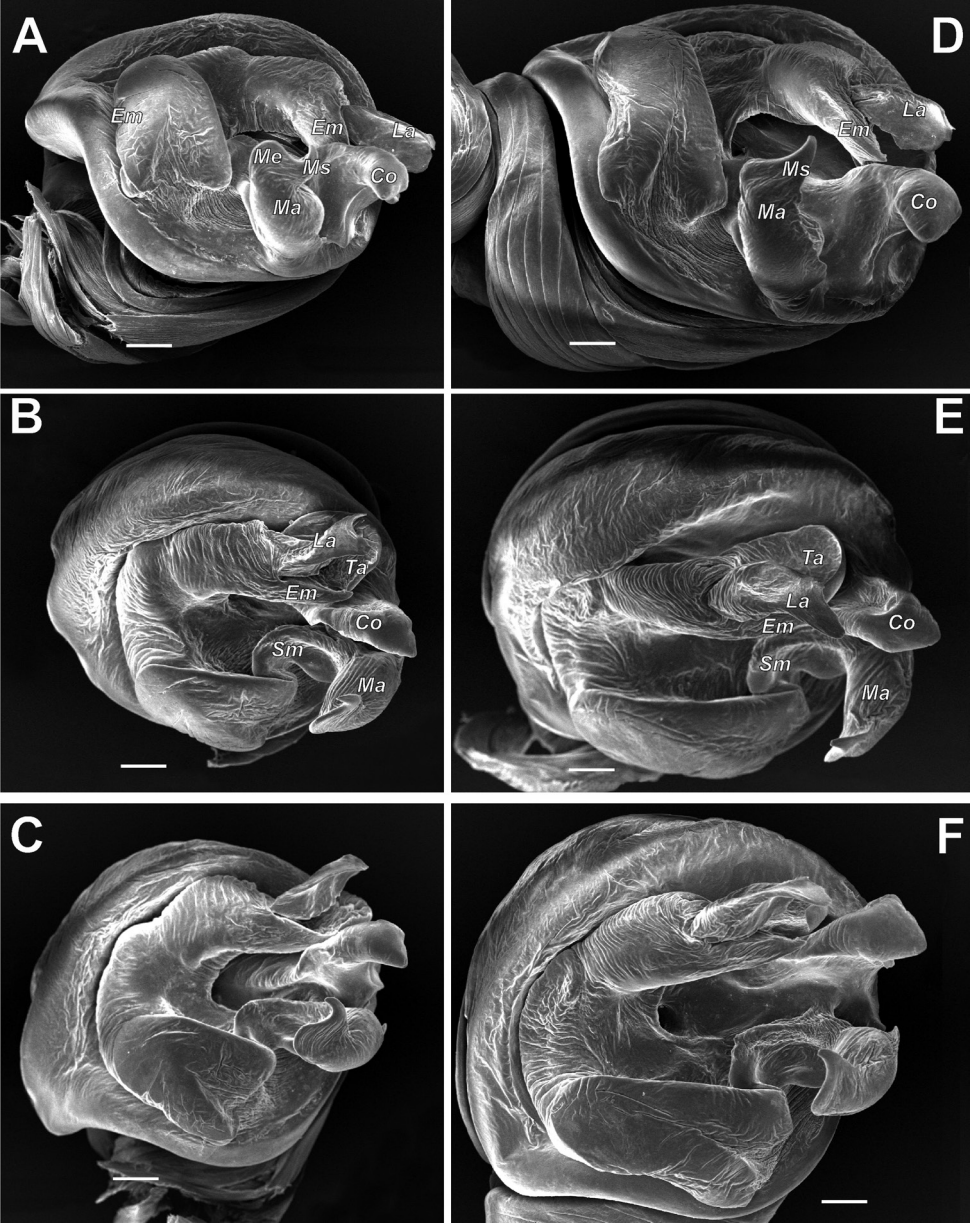
SEM graphs of the bulbs of *Neoscona spasskyi* (**A–C**) and *N. isatis* sp. nov. (**D–F**). **A, C, D, F** Prolateral **B, E** anterior. Abbreviations: *Co* conductor, *Em* embolus, *La* lamella, *Ma* median apophysis, *Ms* spur of median apophysis, *Sm* stipes of median apophysis, *Ta* terminal apophysis. Scale bars: 0.1 mm.

**Figure 17.**
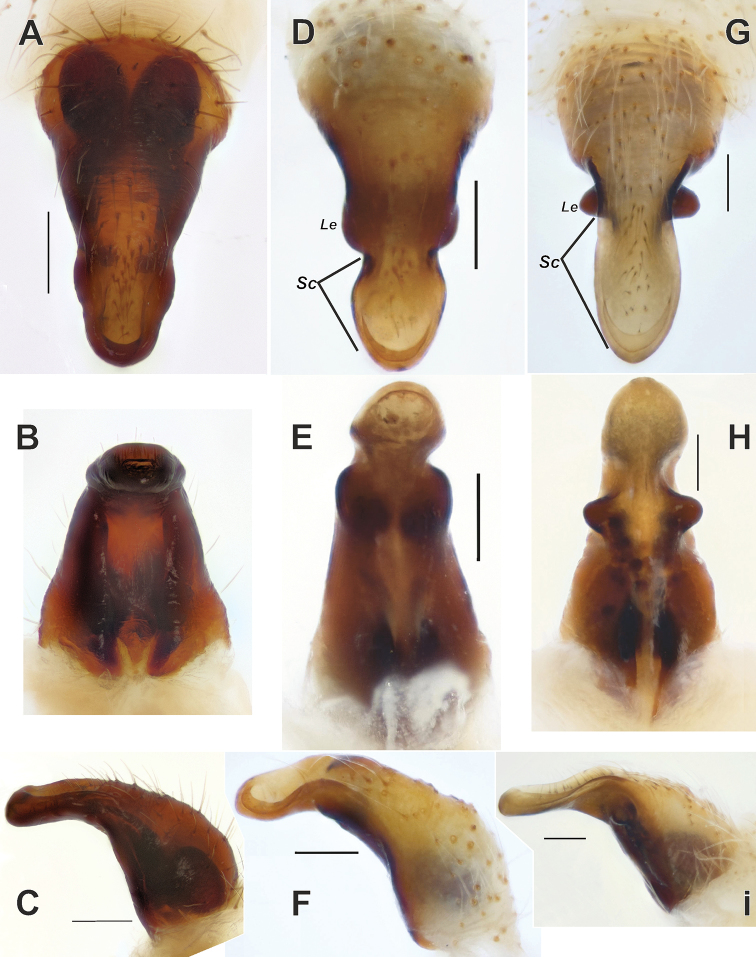
Epigynes of *Neoscona theisi* (**A–C**), *N. spasskyi* (**D–F**) and *N. isatis* sp. nov. (**G–I**). **A, D, G** Ventral **B, E, H** posterior **C, F, I** lateral. Abbreviations: *Le* lateral extension, *Sc* scape. Scale bars: 0.2 mm.

**Figure 18.**
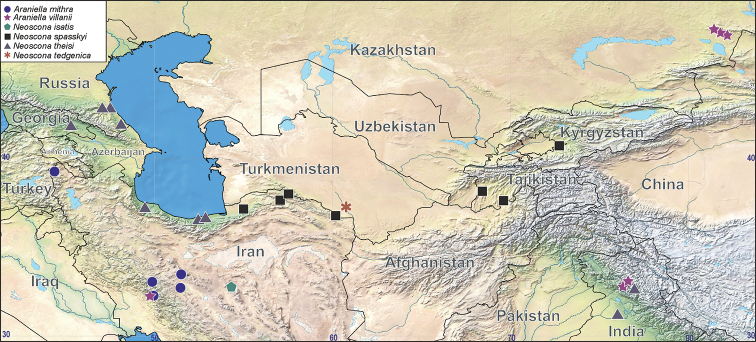
Distribution records of *Araniella mithra* sp. nov. (blue circle), *A. villanii* sp. nov. (violet star), *Neoscona isatis* sp. nov. (green pentagon), *N. spasskyi* (black square), *N. theisi* (gray triangle, only new records) and *N. tedgenica* (brown asterisk).

##### Diagnosis.

*Neoscona spasskyi* differs from the similar *N. theisi* by having a thinner dark median band on the carapace and wider white lateral bands (cf. Figs 11C and 11A, B, D, E). Some specimens of this species have a pyramid-type pattern (Fig. 12A, B) lacking in other species. Males of this species differ from the congeners known in the region by having about 40 ventral spines on tibia II (vs. ca. 90, 20 or 10). *Neoscona spasskyi* differs from *N. isatis* sp. nov. by having prolateral extension of median apophysis. Epigyne of this species has the scape almost as wide as long vs. about twice longer than wide in *N. isatis* sp. nov. It differs from those in *N. theisi* by having distinct constriction (vs. lacking).

##### Description.

**Male.** Habitus as in Figs 11B, 12A. Total length 7.47. Carapace 3.60 long, 2.98 wide in pars thoracica, 1.19 in pars cephalica. Eye sizes and interdistances: AME: 0.20, ALE: 0.14, PME: 0.15, PLE: 0.13, AME–AME: 0.19, PME–PME: 0.12. Carapace, labium, chelicerae, and maxillae reddish brown, carapace with distinct and relatively long foveal mark, slightly darker in submarginal and without any patterns. Sternum with dark frontal edges, and a light median band. Legs the same color as the carapace, with annulations and numerous spines. Abdomen grayish green, dark gray in frontal, and with a distinct dark green patch on dorsum, and two light bands with a dark gray patch between them ventrally. Spinnerets light brown, apical segment lighter. Leg I measurements: 16.48 (5.11, 1.83, 4.11, 4.51, 1.28).

Palp as in Figs 14C, 15A–C, 16A–C. Tegulum without distinct ventral extension; median apophysis (*Ma*) with prolateral extension (*Me*) subequal in length to spur (*Ms*) of median apophysis; stipes of median apophysis (*Sm*) as long as apophysis; lamella (*La*) weakly sclerotized; conductor club-like.

**Female.** Habitus as in Figs 11C; 12B; 13C, D. Total length 8.75. Carapace 3.98 long, 2.97 wide in pars thoracica, 1.50 in pars cephalica. Eye sizes and interdistances: AME: 0.21, ALE: 0.14, PME: 0.15, PLE: 0.13, AME–AME: 0.21, PME–PME: 0.13. Coloration as in male. Leg I measurements: 7.30 (2.08, 1.02, 1.60, 1.75, 0.85).

Epigyne as in Figs 13D, 17D–F. Epigyne with distinct constriction; lateral extensions distinct, wider than long; scape almost as wide as long.

##### Comments.

Types of this species have not been found among the Spassky’s collection in the Zoological Museum, St. Petersburg (Nekhaeva, pers. comm.). Spassky (1952) described this species as *Araneus cruciferoides*, a name preoccupied by Tullgren (1910) on the basis of both sexes. Later, a replacement name, *Araneus spasskyi*, was provided by Brignoli (1983). Marusik et al. (1991) erroneously synonymized it with *Neoscona tedgenica* (Bakhvalov, 1978), a species known only from a female and a juvenile specimen collected in Turkmenistan (Bakhvalov 1978), and transferred to *Aculepeira* by Brignoli (1983). Comparing available figures in Spassky (1952) and Bakhvalov (1978) and the newly studied material, these two species differ in the shape of the posterior scape (rounded vs. triangulate) and the dorsal abdominal pattern (white “true” folium on a dark background in *N. tedgenica*, vs. dark “incomplete” folium on a light background in the other species). For these reasons, we now revalidate the name ‘*spasskyi*’ and establish a new combination for it: *Neoscona spasskyi* (Brignoli, 1972) comb. nov.

##### Distribution.

Tajikistan, Kyrgyzstan (Spassky 1952), Turkmenistan, Iran (first records for both).

#### Neoscona subfusca

Taxon classificationAnimaliaAraneaeAraneidae

(C. L. Koch, 1837)

FC111CC4-9AE6-5834-9125-882DC13BC6F6

Neoscona subfusca : Grasshoff 1986: 15, fig. 2, 4, 11–24 (♂♀); Levy 1998: 336, fig. 96–107 (♂♀).

##### Diagnosis.

This species well differs from other species occurring in the region by the abdomen being as wide as long in the female and with small horns in the male (vs. abdomen longer than wide and lacking horns).

##### Description.

See above-cited literature.

##### Distribution.

Entire Africa, Mediterranean (Grasshoff 1986) to Turkmenistan (Mikhailov 2013).^1^

#### Neoscona tedgenica

Taxon classificationAnimaliaAraneaeAraneidae

(Bakhvalov, 1978)

D82E1654-D841-5D86-BF5A-4E2364F4369C

Fig. 18

Araneus tedgenicus Bakhvalov, 1978: 790, figs 1–4 (♀).
Aculepeira tedgenica : Brignoli 1983: 255.

##### Diagnosis.

*Neoscona tedgenica* differs from the closely similar *N. spasskyi* in the shape of the posterior area of the scape (triangulate vs. rounded) and the dorsal abdominal pattern (white “true” folium on a dark background in *N. tedgenica*, vs. dark “incomplete” folium on a light background in *N. spasskyi*).

##### Comments.

See under *Neoscona spasskyi* (Brignoli, 1983). Types of this species are lost along with the rest of the private collection of Bakhvalov.

##### Distribution.

Turkmenistan (Bakhvalov 1978).

#### Neoscona theisi

Taxon classificationAnimaliaAraneaeAraneidae

(Walckenaer, 1841)*

F0071CFF-B120-51E2-8A5E-683A1F9DE49A

Figs 11A, B ; 12C, D ; 13A, B ; 14A, D ; 17A–C ; 18

Neoscona theisi : Grasshoff 1986: 69, fig. 90–100 (♂♀); Tanikawa 1998: 137, fig. 1–8, 11–17 (♂♀); Tanikawa 2007: 67, fig. 150–159, 572–574 (♂♀).
Neoscona sodom Levy, 1998: 340, fig. 117–126 (♂♀). **syn. nov.**
Neoscona sodom : Bosmans et al. 2019: 9, fig. 1a–e (♂).

##### Material examined.

Iran: 2♂ 5♀ (MHNG), Mazandaran Province: around Amol, 36°18'N, 52°21'E, 18.07.1973 (A. Senglet); 3♂ 6♀ (MHNG), Babol, 36°33'N, 52°42'E, 19.07.1973 (A. Senglet); 5♀ (MHNG), Gilan Province: Rudbar, 36°49'N, 49°25'E, 4.09.1973 (A. Senglet). Russia: Daghestan: 1♂ (PPC), Sergokalinski Dist., Sergokala Vil., 31.07.2008 (A.V. Alieva); 1♀ (PPC), Makhachkala, 08.2009 (S.V. Alieva); 1♀ (PPC), same locality, 08.2008 (S.V. Alieva); 3♀ (PPC), Magaramkentski Dist., Tselegyun Vil., 8.08.2008 (S.V. Alieva); 1♀ (PPC), Kizilyurtovski Dist., Sultan-Yangiyurt Vil., 18.05.2009 (M.A. Aliev, Z.A. Shavlukov); 1♀ (PPC), Karabudakhkentsky Dist., 07.2008. (N.M. Gasanova). Georgia: 1♀ (photographed specimen), Tbilisi, 41.767986N, 44.767779E, 17.09.2019 (A. Seropian). India: 1♀ (MMUE), Himachal Pradesh State: Patlikuhl Town, 32°07'N, 77°08'E, 1200 m, 28–29.5.1999 (Y.M. Marusik); 4♂ 2♀ (MMUE), Punjab State: Patiala, University campus, 30°21'N, 76°27'E, 24–25.6.1999 (Y.M. Marusik); 4♂ 1♀ (MMUE) and 5♀ (MMUE), same data.

##### Diagnosis.

*Neoscona theisi* differs from the congeners occurring in the region by the presence of a wide black median band on the venter of abdomen and thin white lateral stripes (Fig. 12D). Males of this species have tibia II with fewer ventral spines (ca. 20) than *N. spasskyi* (ca. 40) and *N. isatis* sp. nov. (ca. 90) and more than in *N. adianta* (ca. 10). Males of *N. theisi* can be recognized also by the palp with pointed dorsal extension/projection of the tibia (Fig. 14A) (vs. absent), distinct ventral conical projection of the tegulum (*Te*) lacking in other species, broad and well sclerotized lamella and wide conductor (vs. lamella thin and weakly sclerotized, conductor club-like), and long prolateral extension of median apophysis, longer than spur of median apophysis (vs. extension absent or as long as spur). The epigyne of *N. theisi* differs from those of *N. isatis* sp. nov. and *N. spasskyi* by the lack of constriction. Females of *N. theisi* well differ from those of *N. adianta* by having a white median band on carapace, darker abdominal pattern and the epigyne being almost twice longer than wide (vs. white band absent, epigyne almost as wide as long).

##### Description.

See Grasshoff (1986) and Tanikawa (1998).

##### Comments.

*Neoscona theisi* is a widely distributed species, with a current natural range covering Pakistan to Japan. Levy (1998) described *N. sodom* on the basis of both sexes from Israel. Judging by the figures provided in the original description, there are no significant differences in the copulatory organs and habitus of *N. sodom* and *N. theisi*. Therefore, the former name is synonymized with the latter.

##### Distribution.

Pakistan, India, Philippines, China to Indonesia, Japan. Introduced to Seychelles, Pacific Is. (WSC 2019). The westernmost localities of this species (sub *N. sodom*) are Cyprus (Bosmans et al. 2019) and Israel (Levy 1998). New records for Iran, Georgia, and Russia.

## Supplementary Material

XML Treatment for Araniella

XML Treatment for Araniella mithra

XML Treatment for Araniella villanii

XML Treatment for Araniella opisthographa

XML Treatment for Araniella nigromaculata

XML Treatment for Neoscona

XML Treatment for Neoscona adianta

XML Treatment for Neoscona isatis

XML Treatment for Neoscona spasskyi

XML Treatment for Neoscona subfusca

XML Treatment for Neoscona tedgenica

XML Treatment for Neoscona theisi

## Footnotes

For complete list of references see WSC (2019)

For complete list of references see WSC (2019)

For complete list of references see WSC (2019)
